# Alignment of Nutri-Score with Mediterranean Diet Pyramid: A Food Level Analysis

**DOI:** 10.3390/nu14235097

**Published:** 2022-12-01

**Authors:** Antonis Vlassopoulos, Alexandra Katidi, Tereza Savvidou, Maria Kapsokefalou

**Affiliations:** Laboratory of Chemistry and Food Analysis, Department of Food Science and Human Nutrition, Agricultural University of Athens, 11855 Athens, Greece

**Keywords:** Mediterranean diet, Nutri-Score, front-of-pack labelling

## Abstract

The Mediterranean diet (MD) has been incorporated as a healthy diet pattern in food-based dietary guidelines of countries all over Europe and the world. Testing the alignment of Nutri-Score with the MD Pyramid is a key step in ensuring that future food-level policies will not be conflicting with existing dietary guidelines. All foods available (*n* = 4002) in the HelTH database, were classified as eligible or not for inclusion in the MD and they were ranked in their respective tier in the food pyramid following two pyramid schemes, the 1995 traditional and 2020 sustainable MD pyramids. For all foods, Nutri-Score was calculated both as the continuous FSAm-NPS score and its categorical outcome—Nutri-Score grades—and their distribution across the MD pyramid tiers was used as a measure of alignment between the MD and the Nutri-Score algorithm. Only 25% of all foods were eligible under the traditional MD, while the sustainable MD covered ~58% of all foods. For both pyramids, Nutri-Score was successful in clearly separating the foods at the top and the bottom of the pyramids (Nutri-Score Mode “D” or “E” for the top tiers and “A” for the bottom tiers), thus suggesting a good alignment between the two. Good discriminatory capacity was also seen within each tier.

## 1. Introduction

The Mediterranean diet was firstly introduced for its health promoting benefits nearly three decades ago [1] and we recently celebrated a decade of it being recognized as an intangible world heritage monument [2]. In this period, the Mediterranean diet has been promoted across the globe as a healthy dietary pattern and incorporated into multiple policy actions in issues relating equally to health and agriculture/food promotion [3,4,5,6,7].

As research data continue to emerge confirming the role of Mediterranean diet adherence to longevity [8,9], new concepts and metrics are being developed in order to translate dietary guidelines into actionable food and nutrition policy interventions [10]. One such approach is the use of nutrient profiling to develop front-of-pack labeling (FOP) schemes that can effectively guide food choices in line with the existing dietary guidelines [10]. Nutri-Score is one such scheme that converts the nutritional content of foods into a five-tier score ranging from A to E (green to red) from healthier to less healthy choices within food groups [11].

The Nutri-Score scheme has been so far validated through research as a system that could successfully promote longevity and reduce mortality from non-communicable diseases [12,13]. However, more research is needed to understand how this system would operate in the food market and among consumers and, thus its maturity as a tool of public health policy [14].

A great volume of the research is focused on the consumer understanding of Nutri-Score [15] and its ability to discriminate foods successfully based on their nutritional composition [16,17,18]. However, an important element to be studied is whether Nutri-Score grades foods in accordance with the local food-based dietary guidelines in order to assess whether its launch would create confusion among consumers [17,19,20]. Recently, we published an analysis showing that in the Greek marketplace, Nutri-Score showed a good discrimination capacity among foods in the same food group and overall agreement with the national food-based dietary guidelines [21].

However, if Nutri-Score is to be launched regionally it is important to understand how the algorithm performs against not just national guidelines but with the overarching dietary pattern that serves as their inspiration, the Mediterranean Diet itself, and the foods that compose it [22,23,24].

In this study, we aim to assess whether the Nutri-Score algorithm aligns with the Mediterranean Diet guidelines and to what extent. To achieve that, foods currently sold in the Greek marketplace, as curated in the HelTH Branded Food Composition Database (BFCD), were (a) classified as being part of the Mediterranean diet guidelines or not and then (b) their Nutri-Score grading was compared to their positioning in the Mediterranean diet pyramid.

## 2. Materials and Methods

### 2.1. The HelTH Database

Food composition data were extracted from HelTH, a dynamic dataset that compiles data on the nutritional composition and quality characteristics of branded foods available in Greek supermarkets.

HelTH started as an initiative in 2018; its first version (11/2019), used in the analyses herein, provided data for *n* = 4002 food products. In brief, HelTH provides information on the nutritional composition of foods, health and/or nutrition claims made on pack, and information on any other quality claims written on pack (environmental claims, logos, origin, etc.). Data are extracted from food labels available on the e-shops of large supermarket chains in Greece and curated in the database. A detailed description of the methodology and structure of HelTH has been published previously [25]. Herbs and spices, alcoholic beverages, dietary supplements, and foods for special nutritional use were excluded (*n* = 139) as they are not included in the scope of the Nutri-Score according to the European regulation [11].

### 2.2. Nutri-Score Calculation

The latest Nutri-Score algorithm was used in this analysis [26]. In brief, as indicated in the algorithm firstly a continuous score called the FSAm-NPS score was calculated for each food based on their nutritional composition per 100 g/mL of food/beverage [26]. For each food, content of energy (kJ), total sugars (g), saturated fatty acids (SFAs) (g), and sodium (mg) were considered “negative nutrients” and scored from 0–10 with higher scores for higher content. In the case of added fats, instead of SFA content, the ratio of SFA/total fat was used. Protein content (g), fiber content (g), and fruits/vegetables/pulses/nuts/specific oils content (FV%) were considered “positive nutrients” and received points from 0–5 with higher scores for higher content. The FSAm-NPS score ranges from −15 to +40 and was calculated by subtracting the “positive nutrients” score from the “negative nutrients” score. More specifically, fiber and FV scores were subtracted for all products, but the protein score was subtracted only in products with a “negative nutrients” score < 11, those with an FV score > 5 or for cheeses.

Secondly, the continuous FSAm-NPS score was then translated to the categorical five-scale Nutri-Score based on the following criteria [26]: (A) was assigned to solid foods with a score from −5 to −1 or waters, (B) to solid foods with a score from 0 to 2 and beverages from −15 to 1, (C) to solid foods with a score 3 to 10 and beverages from 2 to 5, (D) to solid foods from 11 to 18 and beverages from 6 to 9 and (E) to solid foods from 19 to 40 and beverages from 10 to 40.

All data and information on the nutrient content of each food were based on the label nutritional composition declaration. FV% was estimated based on the ingredient list in a two-step process. Firstly, all foods were screened to assess the presence of at least 40% content in fruits, vegetables, pulses, nuts and rapeseed, walnut, and olive oils, which is the minimum content required. Then, for the products that met this minimum requirement a thorough quantification was carried out.

For the purpose of the study, products that did not contain any data about their energy, saturated fat, total sugar, or sodium content (*n* = 778) were excluded, as no Nutri-Score could be calculated. Missing nutrient values could be due to lack of nutritional declaration or low-quality images obtained from the specific foods. On the contrary for “positive nutrients” missing information was imputed as zero. A detailed analysis of the Nutri-Score calculation in the HelTH dataset has been reported previously [21]. Data imputation as zero was a rare phenomenon in the HelTH database as data completeness for positive nutrients reached >90% for food groups where each positive nutrient was relevant [25].

### 2.3. Alignment with the Mediterranean Diet Guidelines

**Figure 1:**
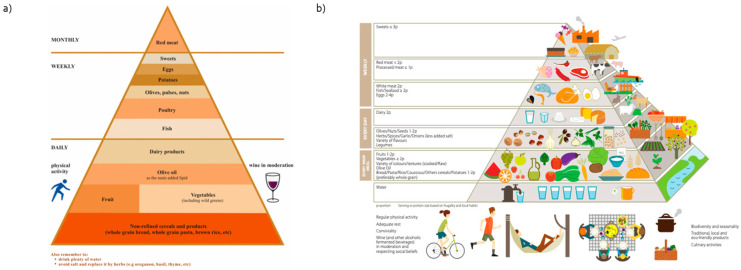
(**a**) The traditional Mediterranean diet pyramid [27] and (**b**) the sustainable Mediterranean diet pyramid [28].

The Mediterranean diet pyramid is the iconic visual summary of the Mediterranean diet guidelines. Two versions of the Mediterranean diet pyramid were used as part of the current analysis, referred to as the traditional or the sustainable Mediterranean diet pyramid. The traditional Mediterranean diet pyramid (tMDP), is the version as originally described based on the Seven Countries study [1,27]. This version is based on the description of the Mediterranean Diet followed by inhabitants of Crete, Greece, primarily in the early 1960s. Thus, it describes a diet based on minimally processed foods, rich in wholegrain cereals, fruits, and vegetables, and composed solely of traditional Greek foods. In particular, according to the tMDP, any novel or modern foods or foods not typically found and produced in Greece were excluded. For example, low-fat Greek cheeses such as low-fat feta or imported cheeses are excluded from the pyramid as are other modern dairies such as yogurt drinks. As shown in Fig1a this pyramid splits foods into 11 tiers from non-refined cereals to red meat with a declining recommended intake both in terms of frequency and number of servings.

The sustainable Mediterranean diet pyramid (sMDP) is the latest update published which was created to expand the Mediterranean diet pyramid as a sustainable dietary pattern for the Mediterranean basin and beyond [28]. In the sMDP, beyond the sustainability considerations, all traditional Mediterranean foods are included as well as a range of modern foods and beverages (e.g., sodas, sweets, savory snacks) which are most often described as foods that fall at the top of the pyramid and hence should be consumed sparingly. The sMDP splits foods into 7 tiers starting from water in the base and going up to fruits, vegetables, and cereals until sweets at the top of the pyramid Figure 1b.

All foods available in HelTH were screened for inclusion in either or both pyramids and then they were assigned to their respective tier according to the specifications of each pyramid. Composite recipes or complex foods that were not clearly described in the pyramids were considered out of scope for the analysis and hence excluded.

### 2.4. Statistical Analysis

Statistical analysis was carried out using IBM SPSS Statistics^®^ (version 23, Northridge, CA, USA). Nutritional composition data (content per 100 g or 100 mL of product) and the FSAm-NPS score were analyzed as continuous variables. Data were tested for normality using the Kolmogorov–Smirnov test. None of the variables followed the normal distribution. Therefore, variables were expressed as median (interquartile range). We assessed the distribution of prepacked products across different Nutri-Score grades for main categories and subcategories and displayed this information in boxplots emphasizing median, 25th, and 75th percentiles. Discriminating ability was considered good when the food group comprised at least three different NS grades [16,19]. Differences were tested using the Kruskal–Wallis non-parametric test for k independent samples. Between-group differences were tested using the Mann–Whitney U test for continuous variables. Statistical significance was set at 0.01% to adjust for multiple comparisons (Bonferroni correction).

## 3. Results

### 3.1. Assignment of Foods in the Mediterranean Diet Pyramids

A total of *n* = 1006 foods (25% of the total) were considered eligible under the tMDP making it the most restrictive pattern. The newly expanded sMDP allowed the inclusion of an additional *n* = 1310 foods, meaning that up to *n* = 2316 foods could be classified as Mediterranean diet compatible under this pattern (57.9% of total) (Table 1). A total of *n* = 1502 foods were classified as modern foods not eligible for inclusion in any version of the Mediterranean diet (37.5%) while a very small proportion of foods (4.6%, *n* = 185) were composite dishes that although traditional and part of the culinary heritage of the Mediterranean could not be mapped in one of the tMDP tiers and were hence excluded.

In both pyramid sweets was the largest food group followed by fruits and vegetables and dairy products. The majority of the additional foods eligible for inclusion in the sMD were assigned to the sweets food group with *n* = 818 foods added compared to the tMDP (*p* < 0.05). The subgroup of cereals was also increased >5-fold under the sMDP, with the inclusion of refined cereal foods (Table 1).

### 3.2. Alignment of Nutri-Score and Mediterranean Diet Pyramid

As far as Nutri-Score performance is concerned the foods included in the tMDP scored better compared to the sMDP foods (FSAm-NPS Score: 4.94 ± 10.5 vs. 8.46 ± 10.5, respectively, *p* < 0.05). When the average FSAm-NPS and Nutri-Score per tier were calculated there was evidence for increasing FSAm-NPS and Nutri-Score from the bottom to the top of both pyramids (Table 2 and Table 3).

In particular for the tMDP, both the non-refined cereal and fruit and vegetable tiers (lowest tiers) received mainly Nutri-Score “A”, on the contrary, red meat and sweets (highest tiers) received mainly Nutri-Score “D”. For the mid-tiers of the tMDP, the Nutri-Score rating did not show a clear pattern as all food groups were either primarily scored as “A” or “B”. As far as the FSAm-NPS score is concerned in the tMD fruits and vegetables received the lowest FSAm-NPS score compared to all other food groups (*p* < 0.001) while on the other hand sweets and red meat received the highest score compared to all other food groups (*p* < 0.001). No differences were observed between the remaining food groups (Table 2).

More linear differences were seen for the Nutri-Score ranking across tiers for the sMDP, with an increase in the primary Nutri-Score ranking from the bottom to the top of the pyramid. The same trend of increasing FSAm-NPS score from the bottom to the top of the sMD pyramid was seen, with the exception of dairy which received a higher score than white meat, fish, and eggs despite being in a lower tier (lower tier represents a recommendation for higher consumption) (Table 3).

A nested analysis within the dairy food group highlighted that there was significant variability in the Nutri-Score performance between dairy subgroups with cheese receiving significantly higher FSAm-NPS scores and Nutri-Score category ranking as compared to milk and yogurt products (*p* < 0.001) Table 4. These differences were mainly driven by the 7-fold and 15-fold higher total fat and sodium content, respectively, despite a 4-fold higher protein content compared to milk and yogurt (data not shown).

## 4. Discussion

This study is the first, to our best knowledge, to directly investigate the agreement of Nutri-Score with the Mediterranean diet pyramid. It expands upon previous work that aimed to measure the alignment of Nutri-Score with national food based dietary guidelines in Mediterranean countries [17,21].

Of the foods analyzed only 25% fit in the traditional Mediterranean Diet Pyramid, and only 57.8% of all foods fit in the updated sustainable Mediterranean Diet Pyramid. Overall, for both pyramids, Nutri-Score was successful in clearly separating the foods at the top and the bottom of the pyramids as the majority of foods at the top were scored as “D” or “E” while those at the bottom received mainly “A”, thus suggesting a good alignment between the two. In the mid of the pyramids, Nutri-Score showed less of a capacity to granularly follow the pyramids as foods in the middle received primarily a score of “B”.

Nutri-Score grades were similar between the two pyramids. As the tMDP represents a more prudent version of the Mediterranean diet the lower average FSAm-NPS score found with this definition is in line with the spirit of the guidelines. When comparisons between the respective tiers are considered the Mediterranean diet definition used did not influence the average FSAm-NPS score in the same or similar tier.

The biggest source of disagreement between the two pyramids was the definitions of tiers and particularly the fact that the tMDP has more tiers/food groups as compared to the sMDP and they are not all positioned in the same level of the pyramid. The most striking differences are those of potatoes and eggs which are mentioned separately from vegetables and white meat, respectively, in the tMDP and positioned higher in the pyramid as opposed to the sMDP. This is linked with an anomaly in the granularity of Nutri-Score grading along the tMDP.

It is of interest to note that all Nutri-Score grades were seen in all tiers, a sign of a good discriminatory capacity as defined in previous research as it can guide towards improvements within the same food group/tier [17,19,21,24]. This became apparent in the nested analysis for dairy products in which it became clear that low-fat, low-sodium dairy such as milk and yogurt receive a substantially better Nutri-Score compared to cheese in line with the Mediterranean Diet guidelines. However, Nutri-Score was still capable of identifying cheese products that were significantly different from their peers, and even within the cheese subcategory, all Nutri-Score grades from “A” to “E” were documented. These results are in line with previous research studying the agreement of Nutri-Score with national food-based guidelines from Mediterranean countries, which reflect more or less the basic principle of the Mediterranean diet pyramid [11,16,17,19,21]. In the case of our analysis, the dataset used did not include any data on vegetable oils [25], as such the results presented herein do not include any data on vegetable oils including olive oil. As per the latest Nutri-Score algorithm [26], olive oil is automatically graded as (C) with discussions underway for an updated automatic “B” grading. Previous research indicates that olive oil is in fact graded better than other fats and oils and that consumers are capable of identifying olive oil as the optimal choice among all fats and oils under the Nutri-Score scheme [21,24].

The current analysis highlighted a challenge linked to the Mediterranean diet pyramid itself and how it affects the mapping of foods as eligible or not. The biggest issue faced in the current analysis was the definition of which foods are considered traditional and local as those are integral to the definition of the Mediterranean diet. For the definition of traditional, a rule of identifying whether a food was part of the culinary tradition in the 1960s was employed for the tMDP, while for the sMDP an extended definition of whether a food would be considered a part of the Mediterranean culinary heritage would be considered [1,27,28]. In both cases, foods produced with modern processing techniques (extrusion, etc.), or including modern ingredients (esp. sweeteners) as well as the low-fat versions of traditional cheeses that were introduced in the market the past two decades (e.g., low-fat feta cheese) were considered as non-eligible. The second element of bias was introduced in the classification of composite foods as part of the Mediterranean diet pyramid. For example, composite traditional dishes such as stuffed vegetables, spinach pie, pulses cooked vegetables, and/or meat, although clearly a part of the culinary heritage of the Mediterranean, was not explicitly mentioned as examples in the Mediterranean diet pyramid guidelines. The classification of such foods within the pyramid remains unclear and difficult, especially for foods combining ingredients from different tiers. To overcome such bias, a systematic approach was developed for the current analysis, and a separate category of traditional composite foods was developed. These foods represented ~5% of the whole dataset and were currently excluded from the analysis. Although they do represent a small proportion of the dataset, it is important to highlight this gap in the Mediterranean diet pyramid guidelines as employing traditional cooking techniques is mentioned as part of the dietary pattern but no consensus is given on how composite foods should be studied and promoted [1,2,28].

At this point, it is important to highlight some limitations of the current study. The primary limitations of this analysis are either linked to the nature of the HelTH BFCD and BFCDs in general or the nature of the Mediterranean diet pyramid guidelines. As a BFCD, HelTH only includes products that are sold as packaged food and those that are required by the legislation to carry a nutritional declaration. Although the use of branded foods data is an improvement in the relevance of the results for the consumer and the food industry as it is a direct reflection of the marketplace as compared to analyses performed on generic food composition data [29,30], for the case of the Mediterranean diet pyramid it also introduces specific hurdles.

In its nature, the Mediterranean diet promotes the consumption of minimally processed, seasonal, and local produce often sold as fresh and non-packaged. A BFCD such as HelTh would not be able to map those products which translate to an underrepresentation of the foods currently available in the marketplace for categories such as fruits, vegetables, meat, and fish. It is safe to stipulate that the majority of packaged foods from these categories will be more extensively processed than the fresh equivalent and that could be linked to an overestimation of the Nutri-Score grade of the respective food category [31].

Despite its limitations, this research is important in starting the discussion around the interplay between Nutri-Score and the Mediterranean diet. As the Mediterranean diet is a building block both for the health and agriculture agenda in the respective countries, ensuring that the two policies are aligned, and act synergistically is key. The current research provides a structured framework to start defining which foods can be considered as part of the Mediterranean diet and whether the available policy options are capable of guiding consumers towards its optimal integration into a healthy diet. An important finding of this work is that depending on the MD definition numerous foods could be excluded from the MD and that highlights the complexity of the MD dietary pattern and the need for in-depth consumer knowledge. It also highlights areas of improvement and future gaps which are necessary to guide future food development. For example, the case of how composite foods are positioned in the Mediterranean diet pyramid and whether the use of modern ingredients and processing techniques should be employed to improve the nutritional quality of traditional products are important considerations for the future. In this light, the current research builds upon previous findings which suggest good agreement between Nutri-Score and the national food-based dietary guidelines [21] by showing good alignment with the Mediterranean diet pyramid as well.

## 5. Conclusions

This study is the first to develop a systematic framework in order to study the alignment of Nutri-Score to the Mediterranean diet pyramid as a guideline to promote healthy diets. It shows that in the larger scheme the Nutri-Score aligns with the Mediterranean diet pyramid and that it successfully separates foods at the two ends of the pyramid. It also shows some capacity to discriminate foods within the same tier of the pyramid based on their nutritional composition, which is an important element of the intended use of the Nutri-Score algorithm.

## Figures and Tables

**Table 1:** Distribution of the foods available in HelTH (*n* = 4002) within the traditional and the sustainable Mediterranean diet pyramid food groups (food groups in the table are not presented according to their ranking in the Mediterranean diet pyramid).

Food Group	Traditional Pyramid *N* (%)	Sustainable Pyramid *N* (%)
Red meat	38 (0.9)	61 (1.5)
Sweets	290 (7.2)	1108 (27.7)
Eggs	32 (0.8)	82 (2.0)
Poultry	n/a	
Fish	47 (1.2)	
Dairy	198 (4.9)	347 (8.7)
Olives, nuts, pulses	133 (3.3)	139 (3.5)
Potatoes	11 (0.3)	579 (14.5)
Fruits and vegetables	184 (4.6)	
Cereals	73 (1.8)	
Total	1006 (100)	2316 (100)

**Table 2:** FSAm-NPS Score and Nutri-Score category across the traditional Mediterranean diet tiers [27].

Pyramid Group	FSAm-NPS Score Mean ± SD	Nutri-Score Category Mode (Range)
Red meat (*n* = 25)	16.68 ± 4.6	D (C–E)
Sweets (*n* = 161)	15.43 ± 6.9	D (A–E)
Eggs (*n* = 27)	−0.63 ± 1.1	B (A–B)
Potatoes (*n* = 11)	−0.63 ± 1.6	B (A–B)
Olives, nuts, pulses (*n* = 118)	1.36 ± 10.5	A (A–E)
Fish (*n* = 34)	5.29 ± 5.6	B (A–D)
Dairy (*n* = 128)	5.23 ± 7.5	A (A–E)
Fruits and vegetables (*n* = 156)	−3.11 ± 8.8	A (A–E)
Non-refined cereals (*n* = 56)	2.48 ± 7.7	A (A–E)

**Table 3:** FSAm-NPS Score and Nutri-Score category across the sustainable Mediterranean diet tiers [28].

Pyramid Group	FSAm-NPS Score Mean ± SD	Nutri-Score Category Mode (Range)
Sweets (*n* = 864)	15.18 ± 7.7	E (A–E)
Red and processed meat (*n* = 44)	16.88 ± 4.0	D (A–E)
White meat, fish, eggs (*n* = 62)	2.94 ± 5.5	B (A–E)
Dairy (*n* = 252)	5.07 ± 7.6	B (A–E)
Olives, nuts, seeds, legumes (*n* = 120)	1.23 ± 10.5	A (A–E)
Fruits, vegetables and cereals (*n* = 465)	−0.39 ± 7.5	A (A–E)

**Table 4:** FSAm-NPS Score and Nutri-Score category among the dairy subgroups.

Food Group	FSAm-NPS Score Mean ± SD	Nutri-Score Category Mode (Range)
Milk (*n* = 95)	0.59 ± 4.3	A (A–E)
Yogurt (*n* = 67)	−0.10 ± 1.8	B (A–C)
Cheese (*n* = 90)	12.62 ± 4.9	D (A–E)

## Data Availability

HelTH is available at https://www.eurofir.org/our-tools/foodexplorer/ (accessed on 27 October 2022).
